# *Plasmodium falciparum* strains spontaneously switch invasion phenotype in suspension culture

**DOI:** 10.1038/s41598-018-24218-0

**Published:** 2018-04-10

**Authors:** Gordon A. Awandare, Prince B. Nyarko, Yaw Aniweh, Reuben Ayivor-Djanie, José A. Stoute

**Affiliations:** 1 West African Centre for Cell Biology of Infectious Pathogens, University of Ghana, Legon, Ghana; 2 Department of Biochemistry, Cell and Molecular Biology, University of Ghana, Legon, Ghana; 3 Department of Biomedical Sciences, University of Health and Allied Sciences, Ho, Ghana; 4 Department of Medicine, Pennsylvania State University College of Medicine, Hershey, PA USA

## Abstract

The extensive redundancy in the use of invasion ligands by *Plasmodium falciparum*, and its unique ability to switch between invasion pathways have hampered vaccine development. *P. falciparum* strains Dd2 and W2mef have been shown to change from sialic acid (SA)-dependent to SA-independent phenotypes when selected on neuraminidase-treated erythrocytes. Following an observation of increasing ability of Dd2 to invade neuraminidase-treated cells when cultured for several weeks, we systematically investigated this phenomenon by comparing invasion phenotypes of Dd2, W2mef and 3D7 strains of *P. falciparum* that were cultured with gentle shaking (*Suspended*) or under static (*Static*) conditions. While *Static* Dd2 and W2mef remained SA-dependent for the entire duration of the investigation, *Suspended* parasites spontaneously and progressively switched to SA-independent phenotype from week 2 onwards. Furthermore, returning *Suspended* cultures to *Static* conditions led to a gradual reversal to SA-dependent phenotype. The switch to SA-independent phenotype was accompanied by upregulation of the key invasion ligand, reticulocyte-binding homologue 4 (RH4), and the increased invasion was inhibited by antibodies to the RH4 receptor, CR1. Our data demonstrates a novel mechanism for inducing the switching of invasion pathways in *P. falciparum* parasites and may provide clues for understanding the mechanisms involved.

## Introduction

As an obligate intracellular parasite, *Plasmodium falciparum* actively invades and establishes successful infection in human erythrocytes, thus, making erythrocyte invasion an attractive target for malaria vaccine development^1^. The invasion process requires interactions between parasite ligands and host cell surface receptors. A major class of erythrocyte surface molecules exploited by *P. falciparum* for invasion are the sialic acid (SA) moieties present on erythrocyte surface glycophorins^2,3^. However, a large proportion of both laboratory and clinical isolates of *P. falciparum* successfully invade SA-deficient erythrocytes^4–9^. Therefore, depending on the requirement of SA for erythrocyte invasion, *P. falciparum* parasites are broadly classified as either SA-dependent or SA-independent. Other erythrocyte receptors that have been shown to be used by the parasite include complement receptor 1 (CR1)^10–12^, basigin^13^, band 3^14–16^, decay-accelerating factor (DAF, CD55)^17^, as well as others yet to be identified^18–22^.

The mechanism of invasion is complicated, and *P. falciparum* deploys a wide repertoire of proteins for interaction with the erythrocyte receptors in a sequence of steps^23–25^. These invasion-related proteins generally belong to two major protein families namely the *P. falciparum* erythrocyte binding antigens (PfEBAs), which include, EBA175, EBA140, EBL1 and EBA181, and the *P. falciparum* reticulocyte binding-like homologues (PfRHs) comprising, RH1, RH2a, RH2b, RH4 and RH5^23^. Redundancy in the roles of the parasite proteins involved in erythrocyte invasion allows the parasite to use the differential expression of these ligands to continuously switch pathways to evade immune recognition and ensure its survival^1,26^. The mechanisms responsible for the switching in gene expression of invasion ligands remain unclear; however, they are thought to be epigenetic, possibly involving histone methylation^27^. Furthermore, the signals that trigger the changes in ligand gene expression are not well-understood, but immune pressure and limiting receptor availability are logical candidates. Much of the current understanding of the ligand switching mechanisms has come from studying two parasite strains, Dd2 and W2mef, which can be induced to switch invasion phenotypes from SA-dependent to SA-independent *in vitro*^28–31^. This switch is known to be normally accomplished by growing the parasites on neuraminidase-treated erythrocytes, which lack SA, and thus induce the parasites to deploy alternative SA-independent pathways^28,30^.

While cultivating Dd2 as a reference for routine invasion phenotyping of clinical parasite isolates in our laboratories, we observed that the parasite’s ability to invade neuraminidase-treated erythrocytes gradually increased over time. This phenomenon appeared to coincide with a change to the use of a shaking incubator for keeping parasite cultures. Therefore, we sought to comprehensively investigate the switching phenomenon by temporally monitoring invasion phenotypes of Dd2, together with the parent strain W2mef, from which Dd2 was cloned, and a commonly used SA-independent strain, 3D7. Gene expression levels of 15 invasion-related proteins, including all the major invasion ligands, were also examined in parasites cultured under static conditions compared to those in suspended cultures. Our results provide a new twist in the complexity of *P. falciparum* invasion mechanisms, and also has interesting implications on the physiological relevance of methods used for parasite cultivation *in vitro* and the study of invasion phenotypes in culture-adapted clinical isolates.

## Results

### *P. falciparum* Dd2 and W2mef spontaneously switch invasion phenotype in suspension cultures

The *P. falciparum* strains Dd2 and W2mef are SA-dependent and therefore their invasion of erythrocytes is ablated upon neuraminidase treatment of erythrocytes^28–30,32^. However, both parasite strains are capable of switching invasion phenotype when continuously selected on neuraminidase (Nm)-treated erythrocytes^28,30^.

**Figure 1:**
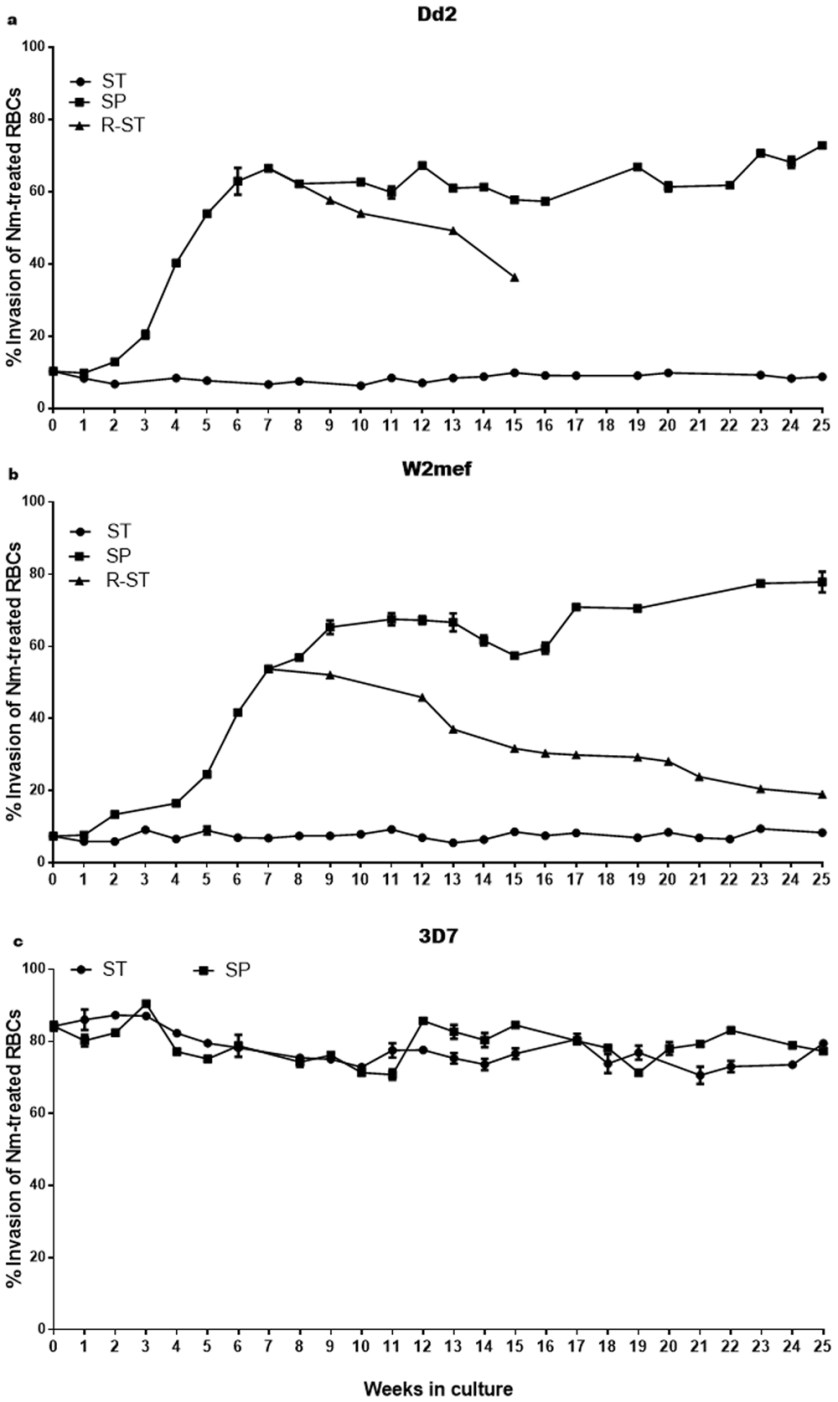
Dd2 and W2mef, but not 3D7, spontaneously switch invasion phenotype in suspension culture. The *P. falciparum* isolates were adapted to parallel *Static* (ST) and *Suspended* (SP) cultures (44 rpm) and their invasion phenotypes assessed weekly using untreated and neuraminidase (Nm)-treated erythrocytes as target cells. Returning SP cultures to ST conditions (R-ST), led to a gradual loss of their ability to invade Nm-treated erythrocytes. Invasion rates were determined by Flow Cytometry as percentage of ring-infected erythrocytes after approximately 14 hours’ incubation of schizonts with target erythrocytes, and expressed as percent invasion efficiency relative to invasion of untreated erythrocytes. Data are presented as mean ± standard errors of triplicate biological experiments.

To investigate the switching of invasion phenotype by Dd2 parasites cultured in suspended conditions, aliquots of Dd2, W2mef, and a commonly used SA-independent strain 3D7, were thawed and split equally into two flasks, one of which was kept in a static incubator (*Static* culture, ST), and the other placed in a gently shaking incubator (*Suspended* culture, SP). After 16 weeks of continuous culture, Dd2 ST and W2mef ST maintained a SA-dependent invasion phenotype, with less than 10% invasion efficiency in neuraminidase-treated erythrocytes (Fig. 1a and b). On the other hand, we observed a dramatic increase in invasion of neuraminidase-treated erythrocytes by Dd2 SP and W2mef SP, beginning from about 20% efficiency at week 3 and peaking at >60% after 6–8 weeks (Fig. 1a and b), indicating a switch to SA-independent invasion phenotype. In contrast, invasion efficiency of the 3D7 strain remained essentially unchanged in both ST and SP cultures over the entire duration of the experiment, although fluctuating within 10% variation (Fig. 1c). Remarkably, when the Dd2 and W2mef SP cultures were taken off the shaker and returned to static conditions (R-ST), the parasites gradually lost their ability to invade neuraminidase-treated erythrocytes and appeared to revert to a SA-dependent phenotype (Fig. 1a and b).

To determine if the Dd2 and W2mef cultures were contaminated with 3D7 or another SA-independent parasite strain, we sought to confirm the genetic identities of both *Static* and *Suspended* cultures of all three strains. Using the highly polymorphic merozoites surface protein 2 (MSP2) and previously described procedures^33–35^ we confirmed that *Suspended* cultures of Dd2, W2mef and 3D7, were genetically identical to their respective *Static* cultures (Supplementary Figure S1). Furthermore, we found that erythrocyte surface expression levels of SA and CR1 were not significantly altered due to shaking (Supplementary Figure S2a and b). Taken together, these data demonstrate that if cultured continuously with shaking, Dd2 and W2mef parasite strains could spontaneously switch to SA-independent invasion phenotype, while maintaining their genetic identity.

### Growth rates of *Static* and *Suspended* parasites in neuraminidase-treated erythrocytes

**Figure 2:**
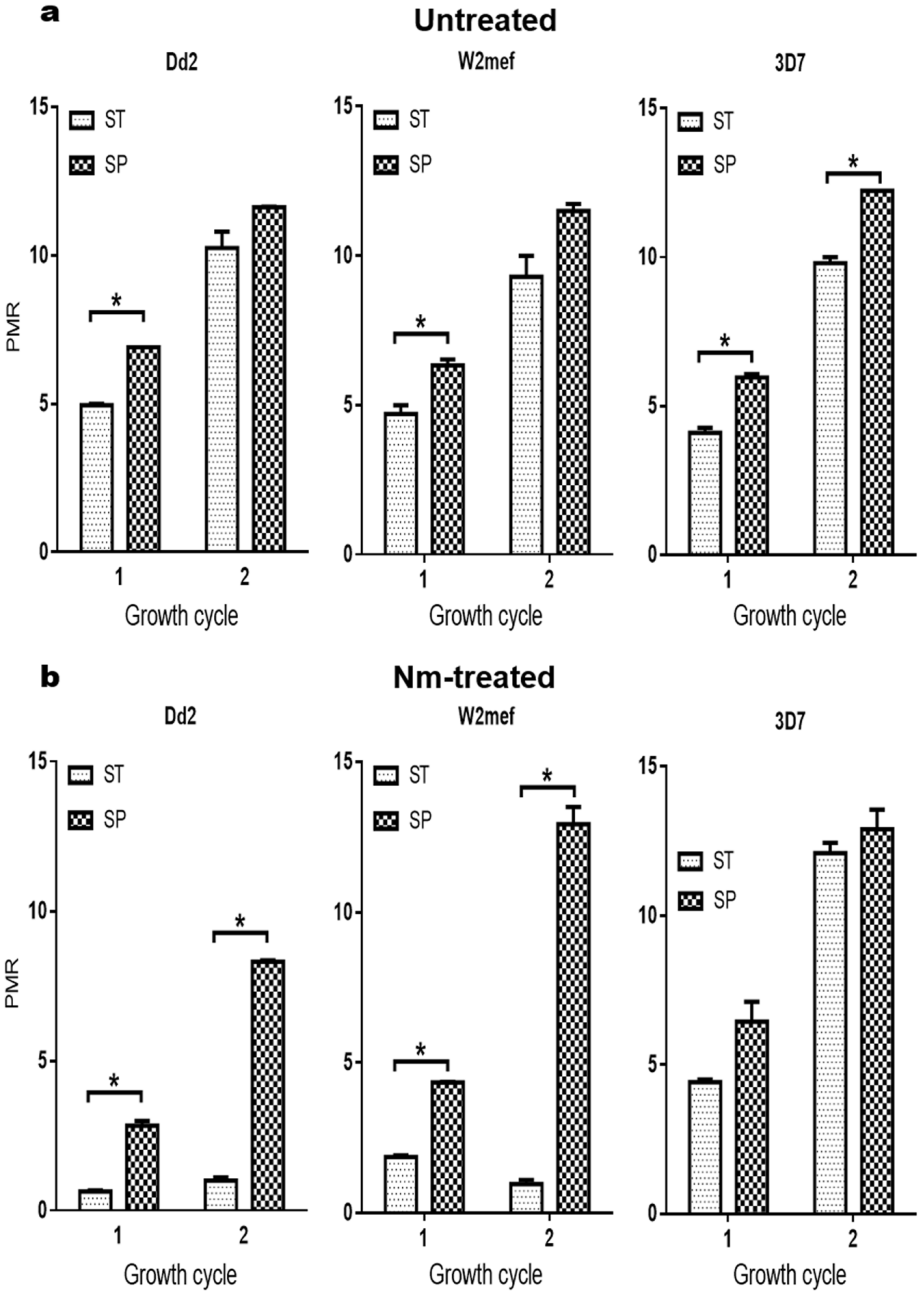
*Suspended*, but not *Static* Dd2 and W2mef, establish successful infection in Nm-treated erythrocytes. *Static* (ST) and *Suspended* (SP) parasites were cultivated in normal (**a**) and Nm-treated (**b**) erythrocytes for 2 asexual cycles. Parasitemias were determined by flow cytometry as percentage of ring-infected erythrocytes at the end of each cycle. Parasite multiplication rates (PMR) were estimated by dividing the parasitemia at the end of each cycle by the starting parasitemia. Data are presented as mean ± standard errors of 2 independent experiments. *P-value < 0.05 at 95% CI; Student t-test.

High multiplication rates, and thus high parasite densities, are virulence features of *P. falciparum* infections^36^. Cultivation of *P. falciparum* parasites in suspended cultures is known to favour a higher rate of multiplication^37–39^. Therefore, it was of interest to determine if the change in invasion phenotypes observed when Dd2 and W2mef were maintained in a shaking incubator was also associated with an increase in growth rate. Furthermore, we sought to establish that *Suspended* Dd2 and W2mef can thrive in Nm-treated erythrocytes, in a similar manner as Dd2NM^28^. Thus, ring stage parasites from 12-week *Static* and *Suspended* cultures were incubated with Nm-treated erythrocytes for two complete erythrocytic parasite cycles, under *Static* and *Suspended* conditions, respectively. Parallel cultures with normal untreated erythrocytes were established as references. Generally, *suspended* parasites grew better than *Static* parasites in untreated erythrocytes (Fig. 2a). However, statistical significance was only achieved at the end of cycle 1 for Dd2 and W2mef (cycle 1: *P* = 0.007 and *P* = 0.045 for Dd2 and W2mef, respectively; cycle 2: *P* = 0.1302 and *P* = 0.0965 for Dd2 and W2mef, respectively). In 3D7 however, the growth rate in *Suspended* parasites was higher than in *Static* cultures at the end of both cycles (*P* = 0.012 and 0.007, respectively). In the Nm-treated erythrocytes (Fig. 2b), *Static* Dd2 and W2mef seemed incapable of establishing successful infections, with multiplication rates of about 1.0. (PMR = 1.01 and PMR = 0.96 at the end of cycle 2 for Dd2 and W2mef, respectively). In contrast, *Suspended* Dd2 and W2mef successfully thrived and established efficient infections (PMR = 8.31 and PMR = 12.93 at the end of cycle 2 for Dd2 and W2mef, respectively), with growth rates well above those in *Static* cultures (*P* < 0.006 and 0.002 for Dd2 and W2mef, respectively). Both *Static* and *Suspended* 3D7 grew normally in Nm-treated erythrocyte (PMR = 12.10 and 12.89 for ST and SP, respectively, at the end of cycle 2), with no statistically significant differences between ST and SP (*P* = 0.403). Our results clearly demonstrate that *Suspended* parasites are capable of establishing successful infections in SA-deficient erythrocytes, confirming that these parasites are SA-independent.

### Sensitivity of *Static* and *Suspended* parasites to neuraminidase, chymotrypsin and trypsin treatment of erythrocytes

**Figure 3:**
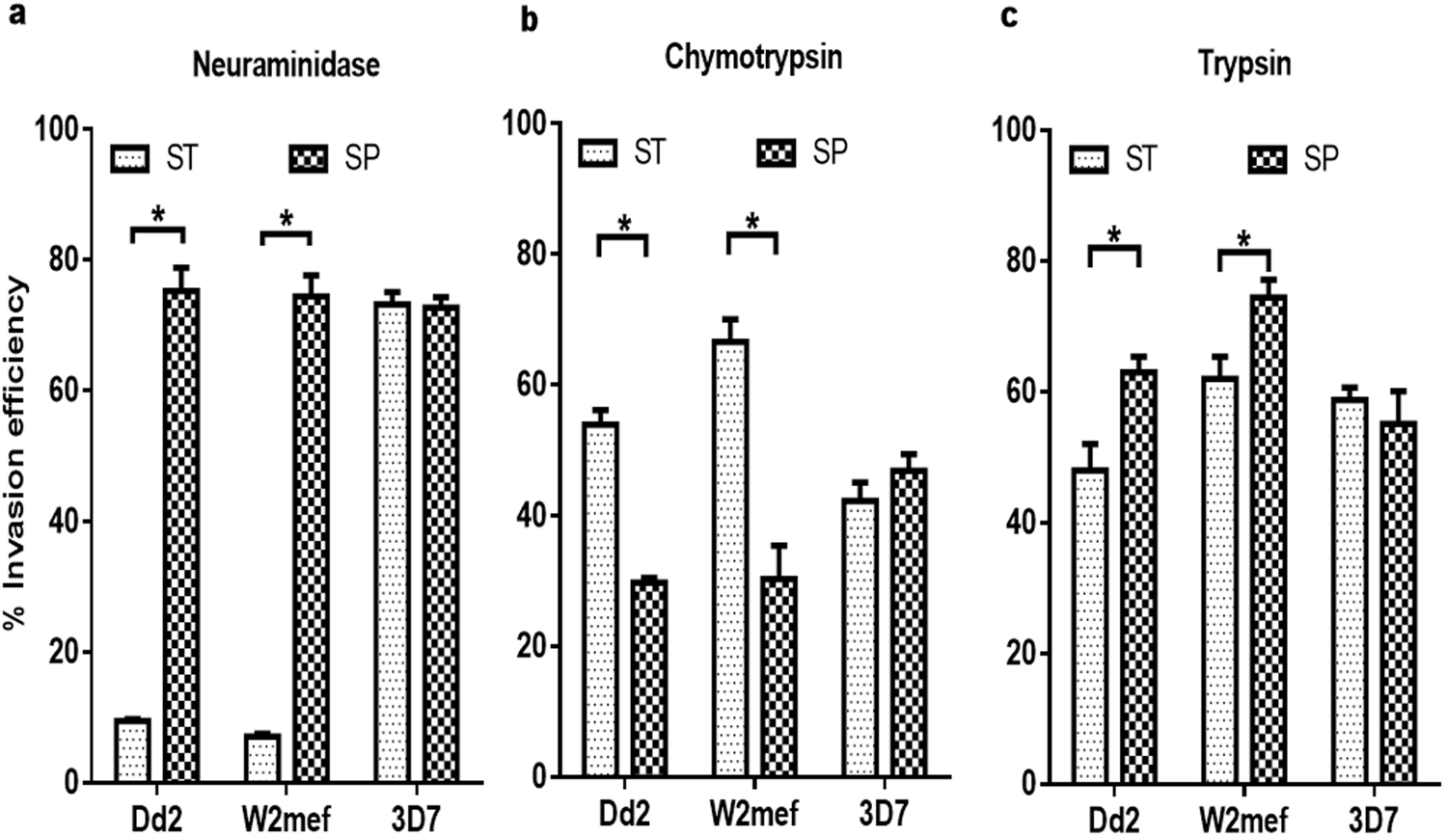
Differential sensitivities of *Static* and *Suspended* parasites to enzyme treatment of erythrocytes. Invasion efficiencies of *Static* (ST) and *Suspended* (SP) into erythrocytes treated with neuraminidase (250 mU), chymotrypsin (1 mg/ml) and trypsin (1 mg/ml) were compared for *P. falciparum* Dd2, W2mef and 3D7 strains. Invasion rates were determined by flow cytometry as percentage of ring-infected erythrocytes after approximately 14 hours’ incubation of schizonts with target erythrocytes, and expressed as invasion efficiency relative to invasion of untreated erythrocytes. Data are presented as mean ± standard errors of three independent experiments performed in triplicates. *P-value < 0.05 at 95% CI; Student t-test.

To determine the relative contributions of the major receptors to the invasion of *Suspended* cultures, parasites were incubated with erythrocytes treated with Nm, chymotrypsin or trypsin, which are enzymes that selectively remove receptors from the cell surfaces. Trypsin usually cleaves glycophorins A and C, but not B, while chymotrypsin cleaves glycophorin B and band 3^40–42^. CR1 is sensitive to both trypsin and chymotrypsin^40^, while basigin is resistant to both enzymes^13,40^. *Static* Dd2 and W2mef parasites were highly sensitive to Nm treatment, with invasion of enzyme-treated erythrocytes reduced to less than 10% relative to untreated erythrocytes (Fig. 3a). Consistent with the switch in phenotype, *suspended* parasite cultures of Dd2 and W2mef were resistant to Nm treatment (Fig. 3a), with invasion reduced to only 75% relative to untreated erythrocytes (Fig. 3a). In the 3D7 cultures, there were no differences in the invasion of Nm-treated erythrocytes by both *Static* and *Suspended* parasites (Fig. 3a). Of interest, invasion by *Suspended* W2mef and Dd2 parasites were significantly more sensitive to chymotrypsin treatment compared to their corresponding *Static* cultures, (*P* = *0.004* for W2mef and *P* < 0.001 for Dd2; Fig. 3b). Conversely, invasion by *Suspended* W2mef and Dd2 cultures were slightly more resistant to trypsin treatment compared to the respective *Static* parasites (*P* = 0.046 for W2mef and *P* = 0.034 for Dd2; Fig. 3c). Sensitivities of *Suspended* 3D7 cultures to chymotrypsin and trypsin were not significantly different from the corresponding *Static* cultures (*P* = 0.288 for chymotrypsin and *P* = 0.526 for trypsin; Fig. 3b and c).

### Sialic acid-independent invasion in switched *Suspended* parasites is CR1-dependent

**Figure 4:**
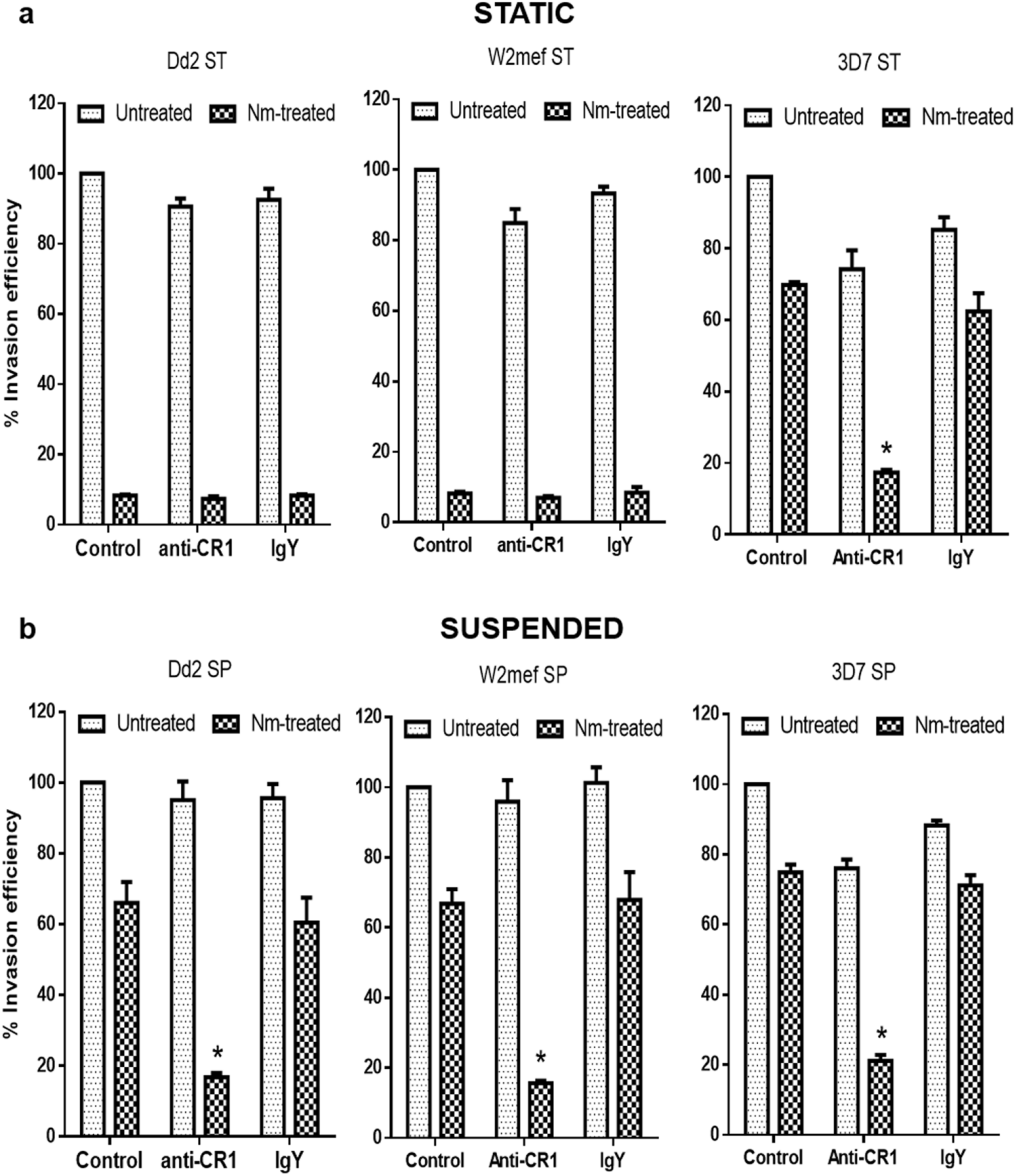
Phenotypic switching of *Suspended* cultures is highly dependent on complement receptor 1 (CR1). Anti-CR1 antibodies (12 µg/mL) inhibited invasion of *Suspended* (SP) parasites into Nm-treated erythrocytes but not untreated erythrocytes. Invasion rates were determined by flow cytometry as percentage of ring-infected erythrocytes after approximately 14 hours’ incubation and expressed as invasion efficiency relative to invasion into untreated erythrocytes. Data are presented as mean ± standard errors of three independent experiments performed in triplicates. *P-value < 0.05 at 95% CI compared to IgY control; student t-test.

The switch in Dd2 and W2mef when cultured on neuraminidase-treated erythrocytes is known to be associated with an increased expression of RH4^30,32^, whose receptor is CR1^10–12^. Therefore, we examined the effect of anti-CR1 antibodies on invasion efficiency of *Static* and *Suspended* cultures into untreated or Nm-treated erythrocytes. Anti-CR1 did not significantly inhibit invasion of any of the parasite strains into untreated erythrocytes compared to isotype control IgY antibodies (*P* > 0.100 for all comparisons; Fig. 4a and b). Furthermore, since *Static* Dd2 and W2mef cultures invade Nm-treated erythrocytes poorly, anti-CR1 antibodies had no significant impact on invasion relative to control IgY (*P* > 0.100 for all comparisons; Fig. 4a). However, for *Suspended* cultures, invasion of both Dd2 and W2mef parasites into Nm-treated erythrocytes was substantially abrogated by anti-CR1 antibodies compared to control IgY (*P* = 0.004 and *P* = 0.003 for Dd2 and W2mef, respectively; Fig. 4b). For 3D7 parasites, invasion into Nm-treated erythrocytes was significantly inhibited by anti-CR1 antibodies compared to IgY for both *Static* and *Suspended* cultures (*P* = 0.001 and P < 0.001 respectively; Fig. 4a and b).

To confirm that these observations were specific to CR1, similar experiments were performed with anti-basigin antibodies, and the results showed that anti-basigin antibodies abrogated invasion of both *Static* and *Suspended* Dd2 and W2mef parasites, and the inhibition was similar in both untreated and Nm-treated erythrocytes (Supplementary Figure S3). Taken together, these data suggest that SA-independent invasion in *Suspended* Dd2 and W2mef cultures is significantly dependent on CR1 receptor usage.

### Specific invasion-related genes are upregulated in *Suspended* parasites

**Figure 5:**
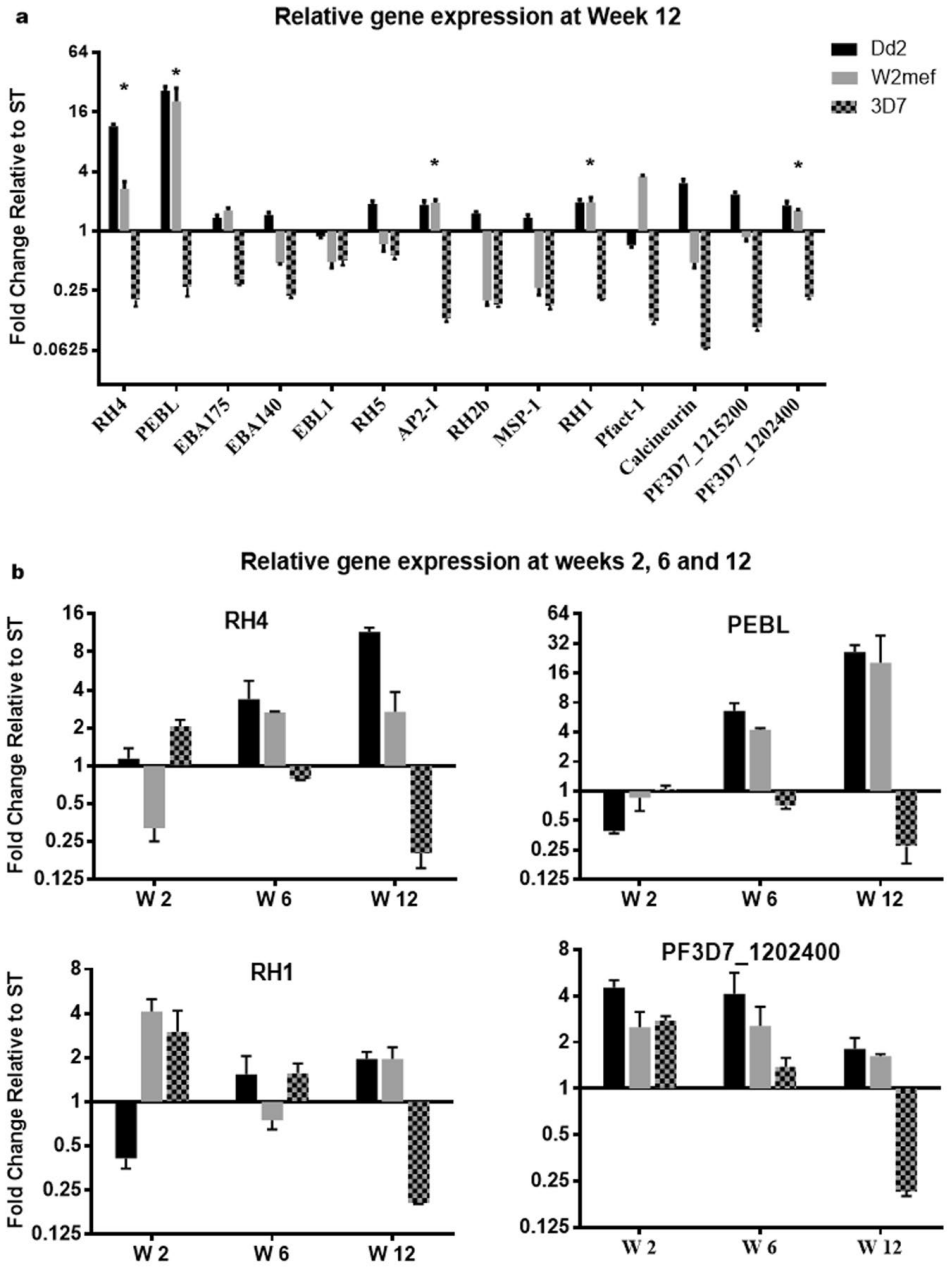
RH4 and PEBL are upregulated in suspended Dd2 and W2mef: Transcript levels of selected *P. falciparum* invasion related genes were determined by reverse transcription quantitative polymerase chain reaction analyses in *Static* (ST) and *Suspended* (SP) Dd2, W2mef and 3D7. The expression level of each gene was normalized to 60S ribosomal L18 protein as an endogenous control and then to AMA1 as a late stage marker. Relative fold changes were determined by normalizing the expression levels in *Suspended* parasites to *Static* parasites. (**a**) Represent transcript levels of all genes at week 12. The transcript level of RH4, PEBL, RH1 and PF3D7_1202400 at weeks (W) 2, 6 and 12 are shown in (**b**). All experiments were run in triplicates. Error bars represent standard errors. *Genes with ≥2-fold change in expression at week 12 in both Dd2 and W2mef.

To investigate the possible mechanisms responsible for the switch in phenotypes, we examined gene expression levels in *Suspended* vs *Static* cultures for a panel of invasion-related genes for which primers were available. Comparison of gene expression levels in *Suspended* Dd2 or W2mef to *Static* cultures after 12 weeks of culture revealed an upregulation and down-regulation of many genes, including RH4, PEBL RH1, RH2b, RH5, actin-1, calcineurin B (CnB), the transcription factor AP2-I and two cyclophilins (PF3D7_1202400 and PF3D7_1215200; Fig. 5a). However, we focused only on genes showing a greater than two-fold change in *Suspended* vs *Static*, and additional temporal expression for these genes was determined, including RH4, PEBL, RH1 and PF3D7_1202400 (Fig. 5b). Expression of all four genes seemed to be upregulated between weeks 2 to 12, particularly for RH4 and PEBL, whose expression remained high at week 12.

## Discussion

The presence of multiple proteins that are functionally redundant is a major hurdle to the development of an effective blood stage vaccine against *P. falciparum* malaria. This functional redundancy has long been attributed to host-immune factors targeting invasion-related proteins^1,26^. However, the potential contribution of non-immune factors to the variation in the expression and use of invasion related genes is poorly characterized. *P. falciparum* Dd2 and W2mef have the unique ability to switch to SA-independent invasion phenotypes when cultured continuously in Nm-treated erythrocytes^28,30^. In addition, deletion of the SA-dependent invasion ligand EBA-175 has also been shown to induce a similar phenotypic switch^29–31^, indicating that this change was driven by limiting access to the SA-dependent invasion pathway. In this study, we have described another remarkable feature of these parasites, which is their ability to spontaneously switch to the SA-independent phenotype, simply by shaking the cultures, without removing SA or its ligands. It was confirmed by flow cytometry that levels of SA molecules on the erythrocyte surfaces were not significantly affected by culturing in suspended conditions. However, the shaking may be increasing the stringency of binding to SA, and the decreased affinity may be mimicking SA removal or EBA-175 deletion, which then triggers the switch in phenotype in the parasite. The potential relevance of this phenomenon *in vivo* is significant, given that parasites in the blood of an infected person are essentially in suspension for a majority of their asexual stage. Thus the dynamics of the parasite phenotypes in peripheral circulation compared to tissue-sequestered ones would be interesting for further investigation.

The phenotypic switch in *Suspended* parasites was accompanied by a substantial increase in sensitivity to chymotrypsin and a modest but significant increase in resistance to trypsin relative to *Static* parasites, which gave a clue about the possible receptors involved. EBA175-deleted W2mef parasites also expressed a similar chymotrypsin sensitivity^29^, which suggests a more significant role for glycophorin B^43,44^ in mediating invasion by *Suspended* compared to *Static* parasites. In addition, the enzyme sensitivity data also implicates CR1, which is resistant to neuraminidase but sensitive to chymotrypsin^40^. Furthermore, a putative RH2b-binding receptor has been characterized as being resistant to trypsin and sensitive to chymotrypsin^41,45^, hence its involvement in mediating erythrocyte invasion by *Suspended* parasites is also possible.

The role of CR1 in mediating SA-independent invasion by *Suspended* parasites was confirmed by receptor inhibition experiments which clearly demonstrated that anti-CR1 antibodies potently abrogated invasion into Nm-treated erythrocytes. These results are consistent with previous observations from us and others that have shown that CR1 is the major SA-independent receptor for both laboratory-adapted parasites and field isolates^5,10–12^. In addition, data presented here also confirmed the critical role played by basigin in mediating erythrocyte invasion by all *P. falciparum* parasites tested to date^5,13^. However, it was clear that the role of basigin was equally important for invasion by *Suspended* and *Static* parasites, suggesting that a role for the RH5-basigin interaction in mediating the phenotypic switch is unlikely.

The ability of Dd2 and W2mef to switch invasion phenotype under selection on Nm-treated erythrocytes or the knock-out of EBA175 has been correlated to the up-regulation of RH4 and PEBL^30,32^. In this study, Suspended Dd2 and W2mef parasites also showed a similar upregulation in RH4 and PEBL gene expression relative to the corresponding *Static* cultures, further confirming that gentle agitation of the parasites over a period of time elicited identical molecular changes to those observed using the other two methods^30,32^. Of potential interest, our studies observed significant upregulation of RH1 and PF3D7_1202400, which have not been previously associated with switching of invasion phenotypes. Thus further investigations are required to determine the potential relevance of this observation to erythrocyte invasion.

It has been demonstrated that the regulation of RH4 expression is through epigenetic mechanisms involving the trimethylation of histone H3K9^27,46^. Our MSP2 genotyping data suggest that there were no changes at the DNA level in *Suspended* parasites compared to the corresponding *Static* parasites, indicating that similar epigenetic mechanisms may be mediating the upregulation of genes in the *Suspended* cultures. Therefore, the relationships between culture agitation and histone methylation will be of significant interest in our ongoing investigations of this spontaneous phenotypic switching phenomenon.

## Materials and Methods

### Parasite culturing

Ethical approval was obtained from the Noguchi Memorial Institute, University of Ghana and all methods used in the study were in accordance with the guidelines and regulations provided by the ethical committee. All human erythrocytes used in this study were obtained with the consent of the donors. *Plasmodium falciparum* strains Dd2, W2mef and 3D7 were cultured in RPMI-1640 (Sigma) supplemented with 0.5% Albumax II (Gibco), 20 mg hypoxanthine, 2 g sodium bicarbonate (Sigma) and 0.05 mg/ml gentamicin sulfate (Sigma) using human group O^+^ erythrocytes at 4% hematocrit in a mixed gas environment of 93% nitrogen, 5% CO_2_, and 2% oxygen (Air Liquide, Birmingham, United Kingdom). Cultures were incubated at 37 °C in either a static incubator (*Static* culture), or with gentle shaking on an orbital shaker rotating at 44 rpm (*Suspended* cultures).

Polymerase Chain Reaction (PCR) was used to confirm the genetic identities of the parasites by amplifying the MSP2 gene from parasite genomic DNA and resolving the amplicons on ethidium bromide-stained 1% agarose gel.

### Enzyme treatment of erythrocyte and invasion assays

Enzymatic treatment of erythrocytes and invasion assays were done as previously described^5^, with few modifications. Briefly, O^+^ erythrocytes were treated with 250 mU/mL of neuraminidase (Nm) from *vibrio cholera* (Sigma Aldrich, St. Louis, Missouri), 1 mg/ml α-chymotrypsin (pretreated with 1-chloro-3-tosylamido-7-amino-2-heptanone, to remove trypsin activity) from bovine pancreas (Sigma Aldrich) or 1 mg/ml trypsin from bovine pancreas (Sigma Aldrich). After incubation for 1 hour at 37 °C with gentle shaking, erythrocytes were washed with RPMI, and subsequently stained with (5-(and-6)-carboxyfluorescein diacetate succinimidyl ester (5(6)CFDA-SE; 20 µM, Invitrogen), a cytoplasmic fluorescent stain, prior to assay plating to differentiate them (acceptor cells) from uninfected erythrocytes in parasite inoculum. Late stage parasites were mixed with acceptor cells in a 1:1 ratio at 2% hematocrit in 96-well titre plates and incubated overnight at 37 °C in a mixed gas environment.

To determine the role of CR1 and basigin (CD147) as receptors for invasion phenotype switching, experiments were set up in the presence of chicken anti-CR1 antibodies or immunoglobulin Y (IgY) control (12 µg/mL, Gallus Immunotech, Fergus, Canada), and mouse monoclonal anti-CD147 antibodies (clone MEM-M6/6; Abcam, USA) or control mouse monoclonal IgG1 (10 μg/mL; Abcam, USA), respectively. All experiments were setup in triplicate in 96-well plates under static conditions and repeated at least once. Plates were incubated overnight (~14 hours) at 37 °C with mixed gas. The cultures were then stained with Hoechst 33342 (5 µM, Sigma Aldrich) after incubation to differentiate parasitized erythrocytes from uninfected ones, and invasion levels determined by flow cytometry (LSR Fortessa X-20, BD) as previously described^5,47^. The percentage of erythrocytes that were dual positive for CFDA and Hoechst 33342 was recorded as the invasion rate.

Erythrocyte surface expression of SA and CR1 was determined in *Static* and *Suspended* cultures by staining with SA-specific mouse anti-human glycophorin A (GPA; clone E4, 0.2 mg/ml, Santa Cruz biotechnology, Inc.) and mouse anti-human CR1 (clone J3D3; 0.1 mg/ml, Santa Cruz biotechnology, Inc.) monoclonal antibodies. Phycoerythrin (PE)-conjugated goat polyclonal anti-mouse antibodies (0.2 mg/ml; ThermoFisher Scienctific) were used as secondary antibodies. Data were analyse with Flowjo software V10.

### Determination of parasite growth rate in untreated and neuraminidase-treated erythrocytes

Parasite cultures were synchronized by treatment with 5% sorbitol to select for ring stages^48^. For growth in untreated erythrocytes parasitemia levels were adjusted to approximately 1% with untreated erythrocytes. For growth in Nm-treated erythrocytes, parasitized erythrocytes were treated with neuraminidase (250 mU) for 1 hour at 37 °C. This was done to prevent SA-dependent reinvasion of uninfected erythrocytes present in parasite inoculum. In addition, uninfected erythrocytes required to sub-culture parasites were also treated with neuraminidase. The parasitemia levels were subsequently adjusted to approximately 1% with the uninfected Nm-treated erythrocytes. All parasitemia levels were confirmed by flow cytometry. The parasites were then cultured for two complete asexual cycles (96 hours). At the end of each cycle, parasitemia were determined by flow cytometry and growth rates calculated by dividing the parasitemia at the end of the cycle by the starting parasitemia.

### Invasion-related gene expression analyses by reverse transcription quantitative polymerase chain reaction (RT-qPCR)

Schizont stage parasites were purified from cultures using the percoll-alanine density gradient centrifugation method. Briefly, parasitized erythrocytes suspended at 50% hematocrit were layered over a percoll-alanine density gradient comprised of 90%, 70% and 40% peroll-alanine (from bottom to top, respectively) in a 15 mL centrifuge tube. Tubes were centrifuged at 2400 rpm for 20 minutes. Late stage parasites at the 70–90% interface were harvested and washed with RPMI. The Schizonts were homogenized with TRIzol Reagent (Ambion/Life Technologies, Carlsbad, California) and total RNA was extracted using the Direct-Zol RNA MiniPrep Plus kit (Zymo Research, USA) according to manufacturer’s protocol. Expression of mRNA transcripts for selected invasion ligands was determined using the Luna Universal One-Step RT-qPCR Kit (New England Biolabs, Inc.), on a QuantStudio 5 Real-Time PCR System (Applied Biosystems), using manufacturer’s recommendations. Data were analyzed with Microsoft Excel and GraphPad software (v.7). Gene expression levels in *Suspended* cultures were expressed as fold-change relative to levels in *Static* cultures, after normalization to the expression of the 60S ribosomal protein L18 as an endogenous control and apical membrane antigen 1 (AMA1) as a parasite maturation marker. Fold-change in gene expression of ≥2 was considered significant.

## Electronic supplementary material

Supplementary Information
