# Supplementary material for: Plasmodium falciparum strains spontaneously switch invasion phenotype in suspension culture
Source: Sci Rep. 2018 Apr 10;8:5782. doi: 10.1038/s41598-018-24218-0 (PMC5893586; doi:10.1038/s41598-018-24218-0)
Supplement: Supplementary file 1 — Supplementary Information [file 41598_2018_24218_MOESM1_ESM.pdf]

Plasmodium falciparum strains spontaneously switch invasion phenotype in suspension culture

Gordon A. Awandare[1,2*ǂ], Prince B. Nyarko[1ǂ], Yaw Aniweh[1], Reuben Ayivor-Djanie[1,3], and Jose A. Stoute[4]

Supplementary data

FIGURE S1

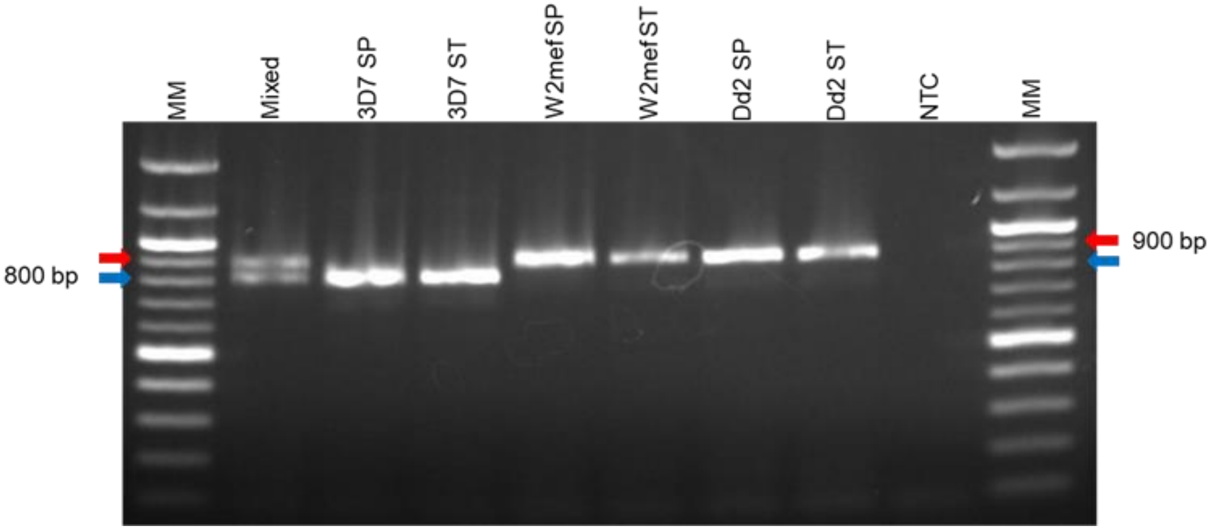

Figure S1: ***Static*** **and** ***Suspended*** **parasites are genetically identical**. A section of the MSP2 gene was amplified from genomic DNA of week 6 parasites. Amplicons were resolved on an ethidium bromide-stained 1 % agarose gel. The primer pair generated amplicons of approximately 800 bp (blue arrow) for 3D7, and 900 bp (red arrow) for Dd2 and W2mef. A combination of all three strains (mixed) showed only two distinct bands of sizes equivalent to 800 bp (3D7) and 900 bp (Dd2/W2mef), respectively. Product sizes were estimated with a 100 bp molecular weight marker (MM). NTC = non template control, ST = *Static*, and SP = *Suspended*.

FIGURE S2

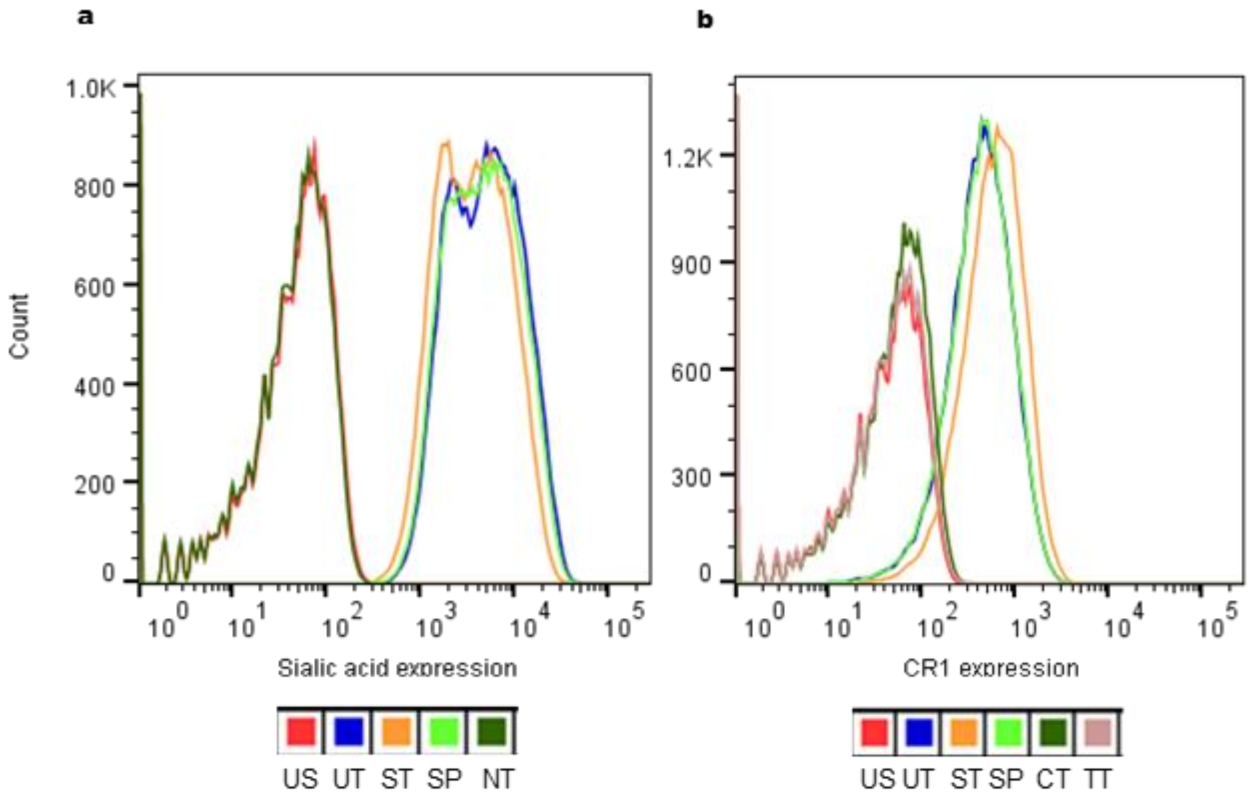

Figure S2: **Shaking does not affect the levels of sialic acid and complement receptor 1 on erythrocyte surfaces**. Erythrocyte surface expression of the invasion receptors (A) sialic acid (SA) and (B) complement receptor 1 (CR1) was determined in *Static* and *Suspended* cultures after two weeks of incubation using flow cytometry. Untreated control (UT), Nm-treated (NT), Chymotrypsin-treated (CT), and trypsin-treated (TT) erythrocytes were stained with mouse monoclonal anti-human GPA antibodies specific to SA or mouse monoclonal anti-human CR1 antibodies. Unstained control erythrocytes (US) were included for comparison. Phycoerythrin (PE)-conjugated goat polyclonal anti-mouse antibodies were used as secondary antibodies. Data were analysed with Flowjo software V10.

FIGURE S3

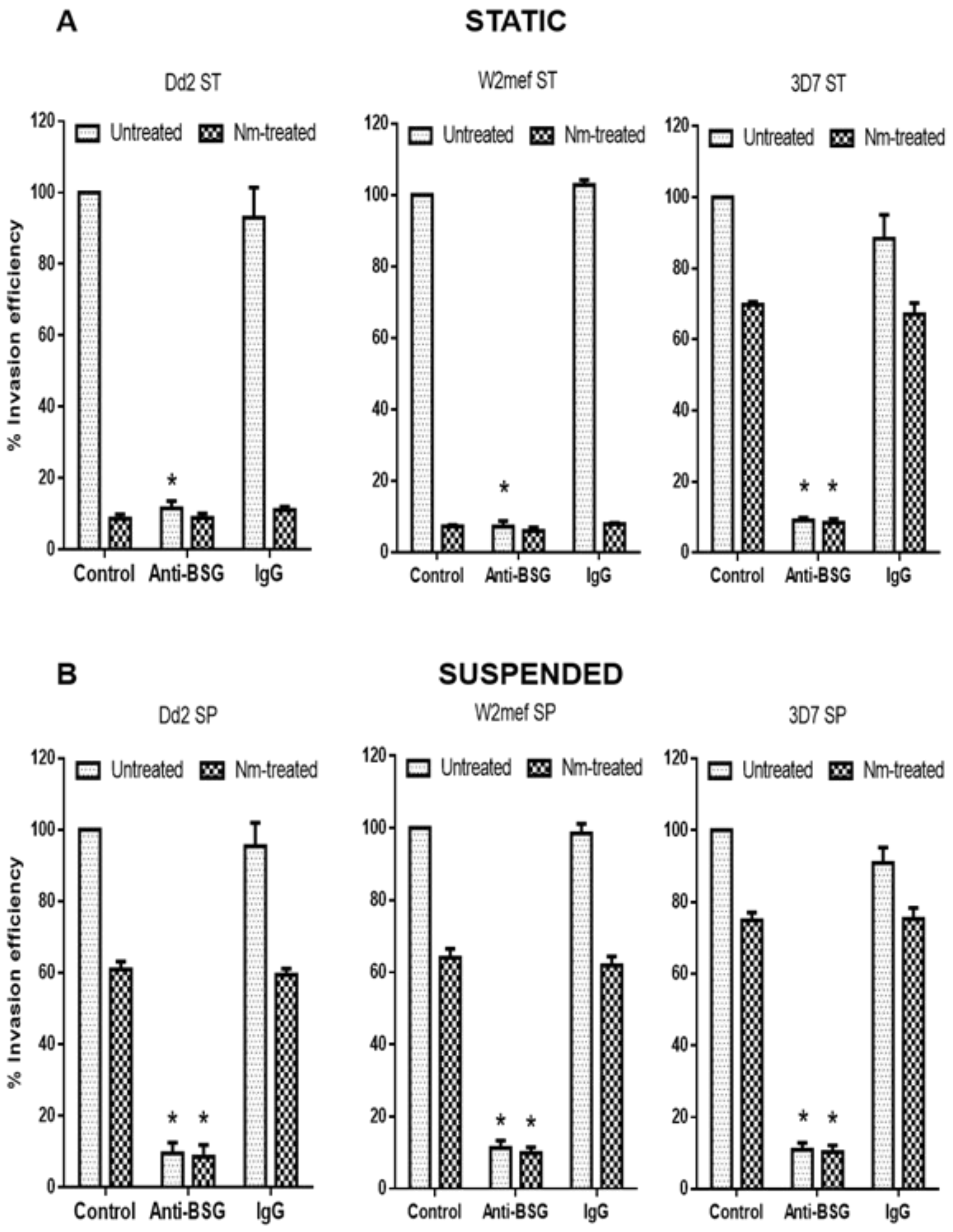
A
STATIC
Dd2 ST
W2mef ST
3D7 ST
Untreated
Nm-treated
% Invasion efficiency
Control
Anti-BSG
IgG
B
SUSPENDED
Dd2 SP
W2mef SP
3D7 SP

Figure S3: Phenotypic switching of *Suspended* cultures not basigin-dependent. Anti-basigin antibodies (10μg/mL) ablated invasion of both *Static* (ST) *Suspended* (SP) parasites into untreated and Nm-treated erythrocytes. Invasion rates were determined by flow cytometry as percentage of ring-infected erythrocytes after 12-16 hours' incubation, and expressed as invasion efficiency relative to invasion of untreated erythrocytes. Data are presented as mean ± standard errors of three independent experiments performed in triplicates. * P-value < 0.05 at 95 % CI compared to IgG control; student t-test.
